# Multi-functional conductive hydrogels based on heparin–polydopamine complex reduced graphene oxide for epidermal sensing and chronic wound healing

**DOI:** 10.1186/s12951-023-02113-9

**Published:** 2023-09-23

**Authors:** Yiyong Dou, Yuwei Zhang, Shuo Zhang, Shuo Ma, Hong Zhang

**Affiliations:** Key Laboratory of Biomaterials of Guangdong Higher Education Institutes, Department of Biomedical Engineering, Jinan University, 510632 Guangzhou, China

**Keywords:** Reduced graphene oxide, Flexible hydrogel sensor, Antibacterial, Anti-oxidative, Chronic wound healing

## Abstract

Flexible hydrogel sensors have expanded the applications of electronic devices due to their suitable mechanical properties and excellent biocompatibility. However, conventionally synthesized reduced graphene oxide (rGO) encounters limitations in reduction degree and dispersion, restricting the conductivity of graphene hydrogels and impeding the development of high-sensitivity flexible sensors. Moreover, hydrogels are susceptible to inflammation and bacterial infections, jeopardizing sensor stability over time. Thus, the challenge persists in designing conductive hydrogels that encompass high sensitivity, antibacterial efficacy, and anti-oxidative capabilities. In this study, GO was modified and reduced via a heparin-polydopamine (Hep-PDA) complex, yielding well-reduced and uniformly dispersed Hep-PDA-rGO nanosheets. Consequently, a hydrogel utilizing Hep-PDA-rGO was synthesized, showcasing commendable conductivity (3.63 S/m) and sensor performance, effectively applied in real-time motion monitoring. Notably, the hydrogel’s attributes extend to facilitating chronic diabetic wound healing. It maintained a suitable inflammatory environment credited to its potent antibacterial and antioxidative properties, while its inherent conductivity promoted angiogenesis. The multifunctional nature of this hydrogel highlight its potential not only as an epidermal sensor but also as a promising dressing candidate for chronic wound treatment.

**Supplementary Information:**

The online version contains supplementary material available at 10.1186/s12951-023-02113-9.

## Introduction

In recent years, flexible sensors have found widespread application in biomedical fields, such as electronic skin, wearable devices, and implantable electronic devices, owing to their remarkable electrical response to external stimuli like temperature, pressure, tension, and vibration [1–3]. The inherent flexibility, exceptional strain tolerance, and excellent conductivity retention of conductive hydrogels during repeated deformation, along with their mechanical properties that match well with biological interfaces, make them extremely promising material for wearable devices and implantable sensors [1, 4]. However, conventional conductive hydrogels used in these applications can trigger inflammatory reactions, wound infections, or immune responses as part of the body’s response to foreign substances [5]. This, in turn, affects the long-term stability of the devices. Consequently, the development of conductive hydrogels that possess high sensing sensitivity, as well as antimicrobial and antioxidative capabilities, continues to present a significant challenge.

Skin tissue is highly sensitive to electrical signals, exhibiting conductivity values ranging from 2.6 to 1×10^−4^ mS/cm [6]. When the skin is damaged, naturally occurring microelectric currents stimulate and regulate cellular behavior, facilitating wound healing and regeneration. In recent years, the use of electrical stimulation (ES) to treat chronic wounds has gained popularity [7]. The ability of ES to restore the weakened endogenous current in chronic wounds provides an incredible opportunity to accelerate wound healing [8, 9]. However, in exogenous ES, electrodes are typically implanted near the wound sites to supply current, rather than covering the entire wound area, which significantly impacts the therapeutic effectiveness. A notable example of chronic wounds is diabetic wounds, which often exhibit delayed healing due to severe bacterial infections, excessive oxidative damage, and prolonged inflammation [10]. Unfortunately, most hydrogel dressings designed for diabetic wounds lack electrical activity and fail to respond to physiological electrical signals at the wound sites, resulting in a passive healing process. Hence, a synergistic therapeutic approach that combines a multifunctional electroactive hydrogel (possessing conductivity, antibacterial, and antioxidative properties) with ES may offer a new avenue for accelerating the healing of diabetic wounds [11–14].

Graphene, known for its exceptional electrical conductivity, holds great promise in the field of biosensors [15]. However, its application as a biomaterial is severely restricted by its tendency to aggregate. Graphene oxide (GO), an oxygenated derivative of graphene, offers improved dispersion but lacks electrical conductivity [16]. Reduced graphene oxide (rGO), on the other hand, possesses a reduced number of oxygen-containing groups and exhibits a balance of conductivity and hydrophobicity, depending on the reduction methods employed [17, 18]. A novel approach for obtaining rGO involves the partial conversion of GO to graphene through dopamine oxidation, inspired by PDA polymerization [19]. This method has led to the preparation of PDA-reduced rGO nanosheets (PDA-rGO), which have been incorporated into scaffolds to fabricate electroactive composite hydrogels [20]. However, the limited reduction degree and inadequate dispersion of PDA-rGO nanosheets have resulted in composite hydrogels with relatively low conductivity and a discontinuous conductive pathway [21]. Consequently, the manufacturing of highly sensitive flexible sensors based on rGO hydrogel has been hindered.

**Scheme 1:**
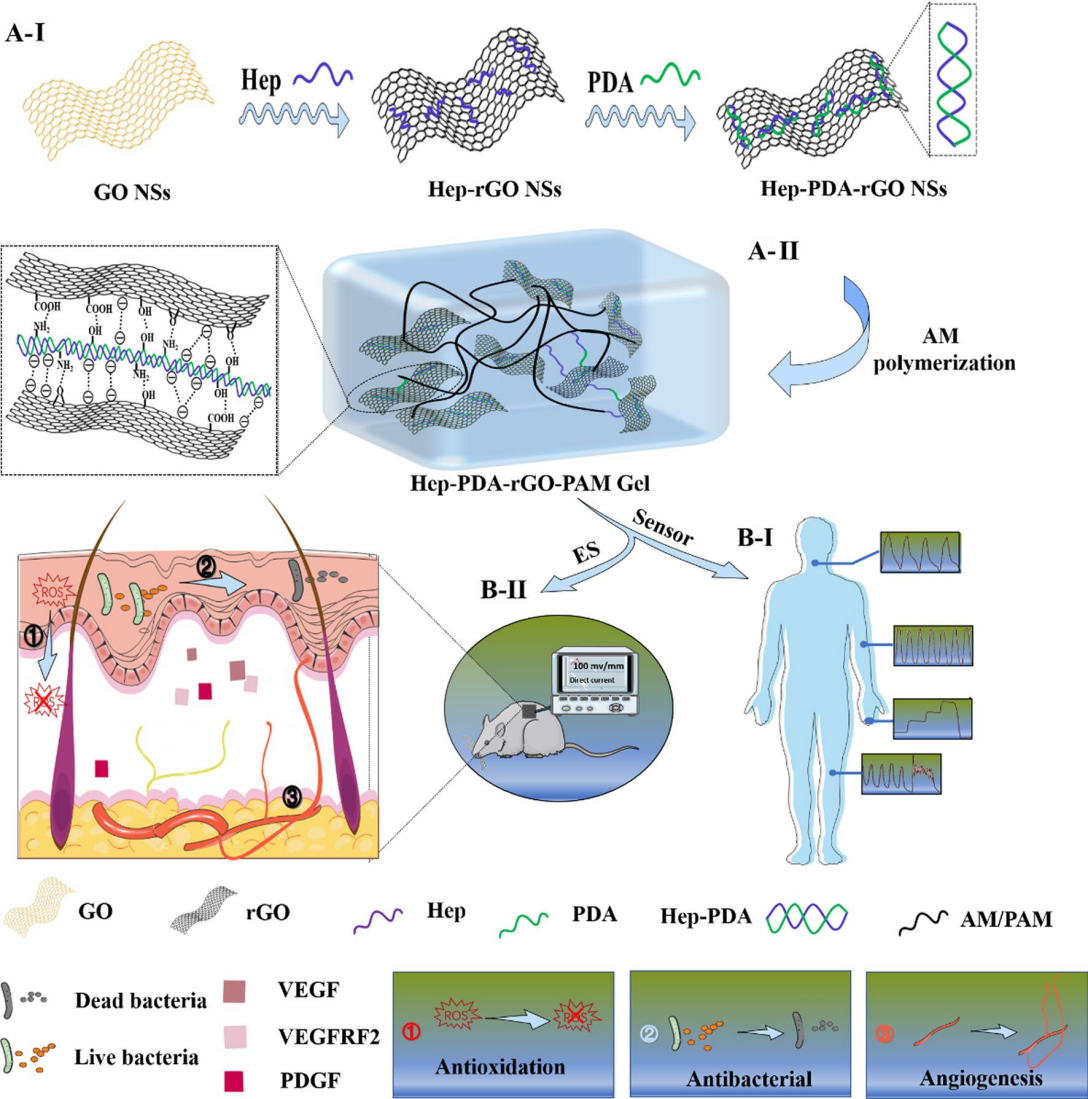
Schematic illustration of the fabrication and application of multifunctional hydrogels. Hep-PDA-rGO nanosheets were synthesized in two steps with improved stability attributed to strong negative repulsion (**A-I**) and then incorporated into a hydrogel matrix to create a conductive Hep-PDA-rGO-PAM gel (**A-II**). The gel was utilized in two applications: as an epidermal sensor (**B-I**) and as a diabetic wound dressing integrated with ES due to its multi-functionalities (**B-II**)

In this study, we incorporated Hep-PDA complex-reduced rGO (Hep-PDA-rGO) sheets into a polyacrylamide (PAM) network to create a versatile conductive hydrogel (referred to as Hep-PDA-rGO-PAM). Our hypothesis was twofold: (i) The Hep-PDA complex effectively reduced GO to rGO with significant reduction while preventing rGO aggregation (Scheme 1A-I). This process yielded the Hep-PDA-rGO-PAM hydrogel, characterized by heightened conductivity and an interconnected electronic transmission pathway (Scheme 1A-II). The hydrogel’s potential for wearable electronic device was demonstrated by real-time monitoring of human motions (Scheme 1B-I). (ii) The Hep-PDA-rGO-PAM hydrogel exhibited robust antimicrobial efficacy against both Gram-negative (*P. aeruginosa*) and Gram-positive (*S. aureus*) bacteria, coupled with enhanced antioxidative prowess, effectively scavenging ROS and mitigating inflammation in diabetic wounds. Moreover, in combination with ES, the multifunctional Hep-PDA-rGO-PAM hydrogel demonstrated enhanced angiogenic therapeutic effects on diabetic wounds (Scheme 1B-II). This work opens up new possibilities for designing versatile rGO-based conductive hydrogels with multifaceted functionalities for both diagnostics and therapies.

## Results and discussion

### Preparation and characterization of Hep-PDA-rGO nanosheets

A facile two-step approach was developed to form the Hep-PDA-rGO nanosheets (Scheme 1A). Firstly, GO was chemically deoxidized by mixing it with heparin to generate heparin-adherent rGO (Hep-rGO). The functionalization of heparin on both sides of the rGO surface occurred through non-covalent interactions, such as hydrophobic and hydrogen bond interactions. The strong negative charge density resulting from the carboxyl and sulfonic groups in heparin prevented the Hep-rGO from aggregating due to electrostatic repulsion [22]. Secondly, Hep-rGO was added to the PDA solution (dopamine was pre-polymerized under oxidative and alkaline condition for 20 mins), and further reduced by PDA for 10 mins to generate Hep-PDA-rGO. After that, the Hep-PDA complex [23] self-assembled through ionic forces effectively immobilized onto the rGO surface, enhancing the stability of the nanosheets. Therefore, the Hep-PDA complex served not only as a strong reducing agent but also as an effective stabilizer for the rGO nanosheets. The composition of each rGO nanosheet is shown in Additional file 1: Table S1.

**Fig. 1:**
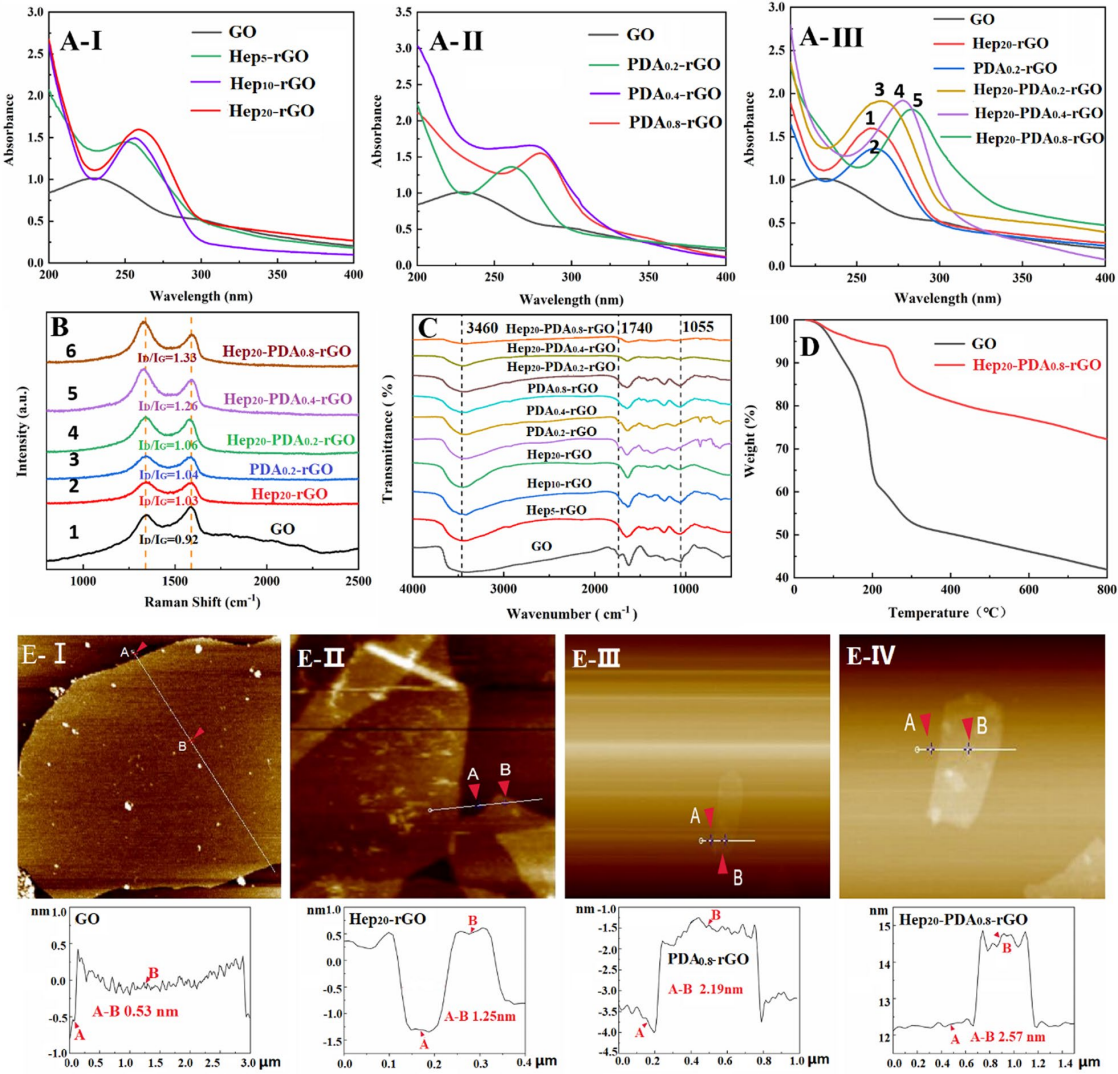
Spectral characterization of GO, Hep-rGO, PDA-rGO and Hep-PDA-rGO: **A** UV–visible absorption spectra, **B** Raman spectra, and **C** FT-IR spectra; **D** Thermal stability of GO and Hep_20_-PDA_0.8_-rGO was evaluated via TGA in the temperature range of 0–800 °C at 10 °C/min; **E** AFM images and the corresponding height measurements of GO (I), Hep_20_-rGO (II), PDA_0.8_-rGO (III) and Hep_20_-PDA_0.8_-rGO (IV). The subscript represents the weight multiples relative to rGO

**Fig. 2:**
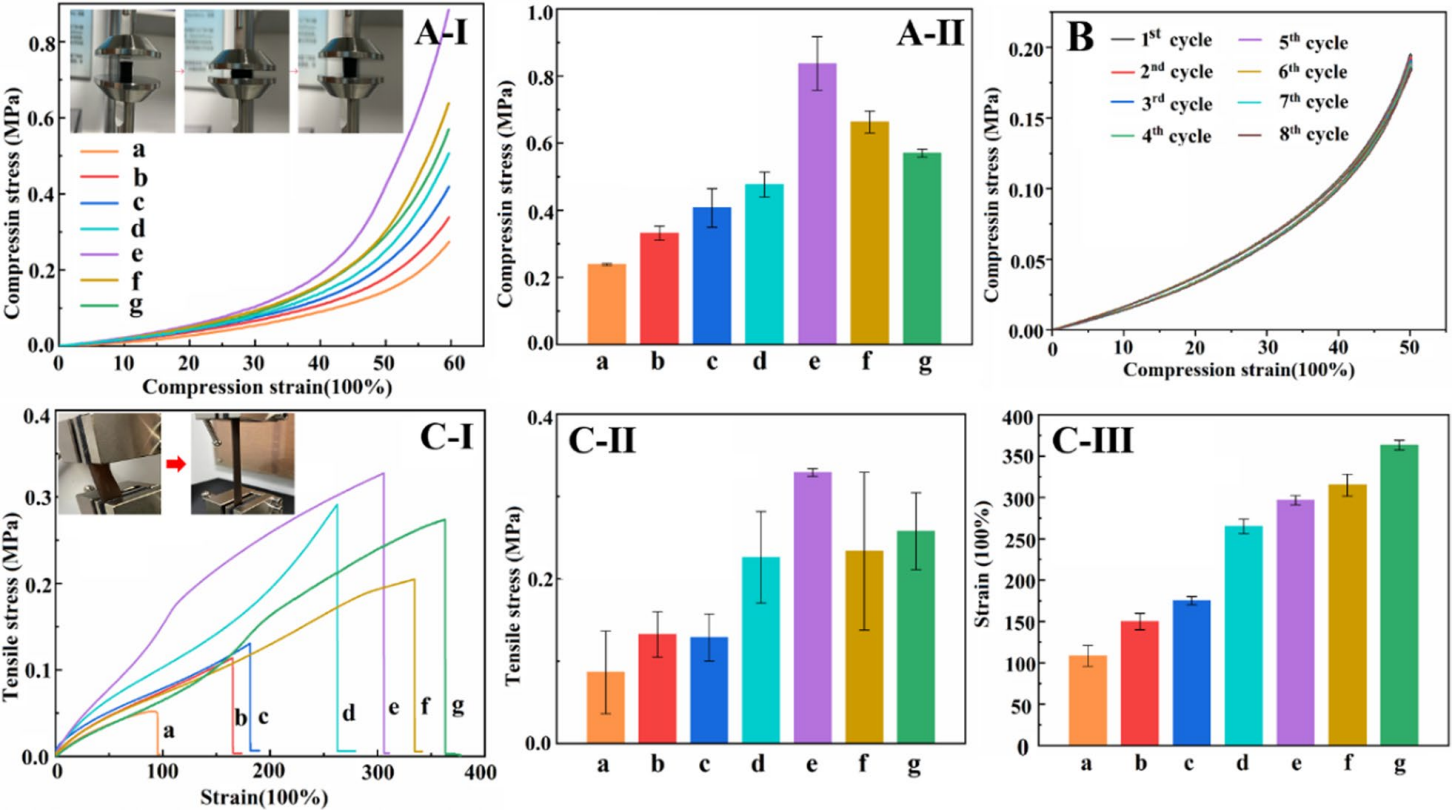
Compressive stress-strain curve (**A-I**) and compressive strength histogram of hydrogel (**A-II**); 8 cycles compressive stress-strain curve of Hep_20_-PDA_0.8_-rGO-PAM hydrogel at 50% of maximum strain (**B**); Tensile stress-strain curve (**C-I**), tensile stress histogram (**C-II**) and tensile strain histograms of the hydrogels (**C-III**) (**a–g** are as follows: (**a**) PAM (**b**) GO-PAM (**c**) Hep_20_-rGO-PAM (**d**) PDA_0.8_-rGO-PAM (**e**) Hep_20_-PDA_0.2_-rGO-PAM (**f**) Hep_20_-PDA_0.4_-rGO-PAM (**g**) Hep_20_-PDA_0.8_-rGO-PAM)

**Fig. 3:**
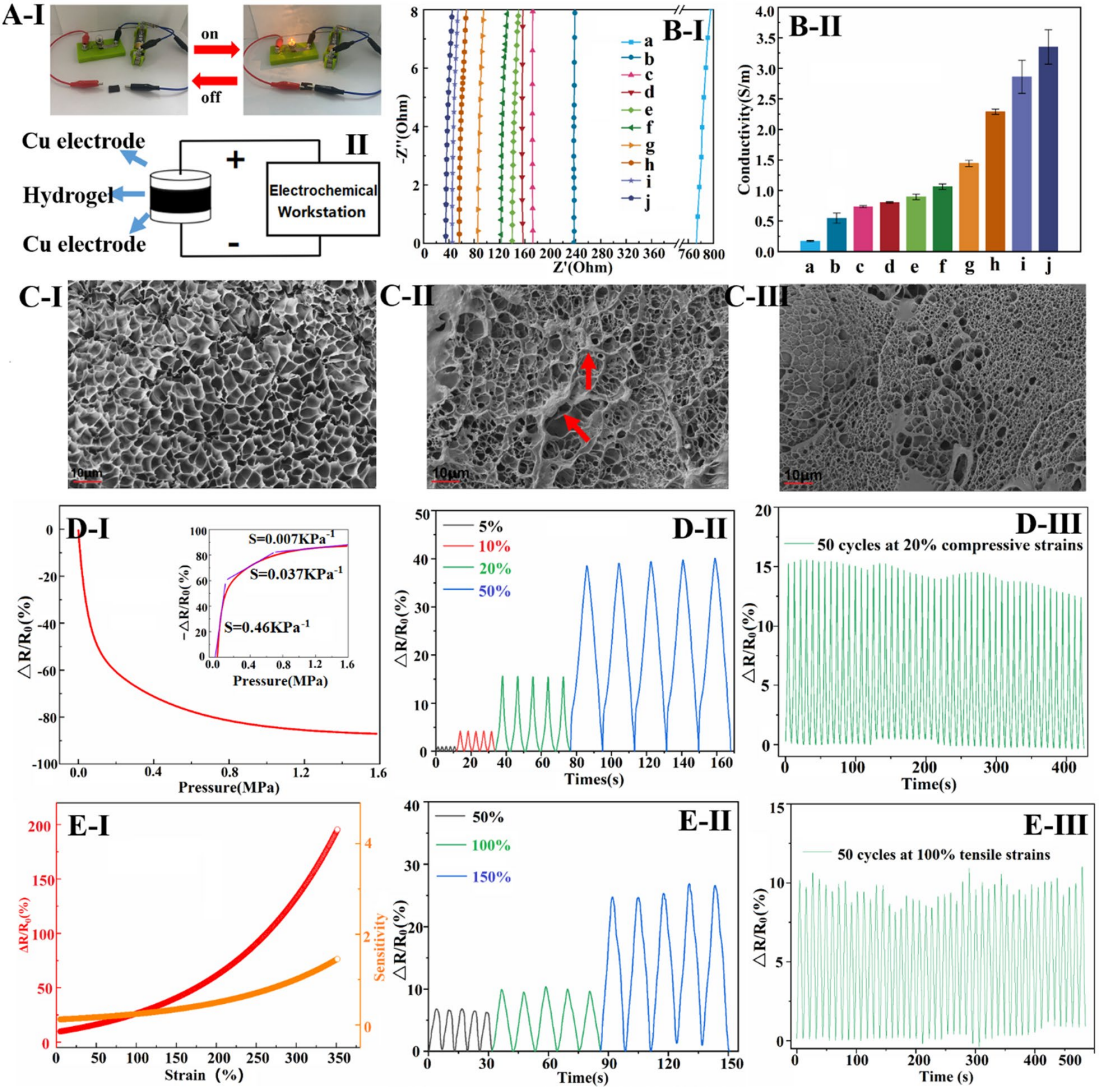
Diagram showing conductive hydrogels connected to a circuit with a lighted bulb (**A-I**) and schematic diagram of the hydrogel conductivity test (**A-II**); Nyquist plots (**B-I**) and conductivity histograms (**B-II**) of different conductive hydrogels; Microscopic morphology of a (**C-I**), g (**C-II**) and j (**C-II**) hydrogels (**a–j** are as follows: (**a**) GO-PAM (b) Hep_5_-rGO-PAM (**c**) Hep_10_-rGO-PAM (**d**) Hep_20_-rGO-PAM (**e**) PDA_0.2_-rGO-PAM (**f**) PDA_0.4_-rGO-PAM (**g**) PDA_0.8_-rGO-PAM (**h**) Hep_20_-PDA_0.2_-rGO-PAM (**i**) Hep_20_-PDA_0.4_-rGO-PAM (**j**) Hep_20_-PDA_0.8_-rGO-PAM); ΔR/R_0_ and sensitivity of conductive hydrogels under compressive stress (**D-I**) and tensile strain (**E-I**); ΔR/R_0_ for 5 cycles at different compressive stress (**D**-**II**) and tensile strains (**E**-**II**); ΔR/R_0_ at 20% compressive strain (**D-III**) and 100% tensile strain for 50 cycles (**E**-**III**)

**Fig. 4:**
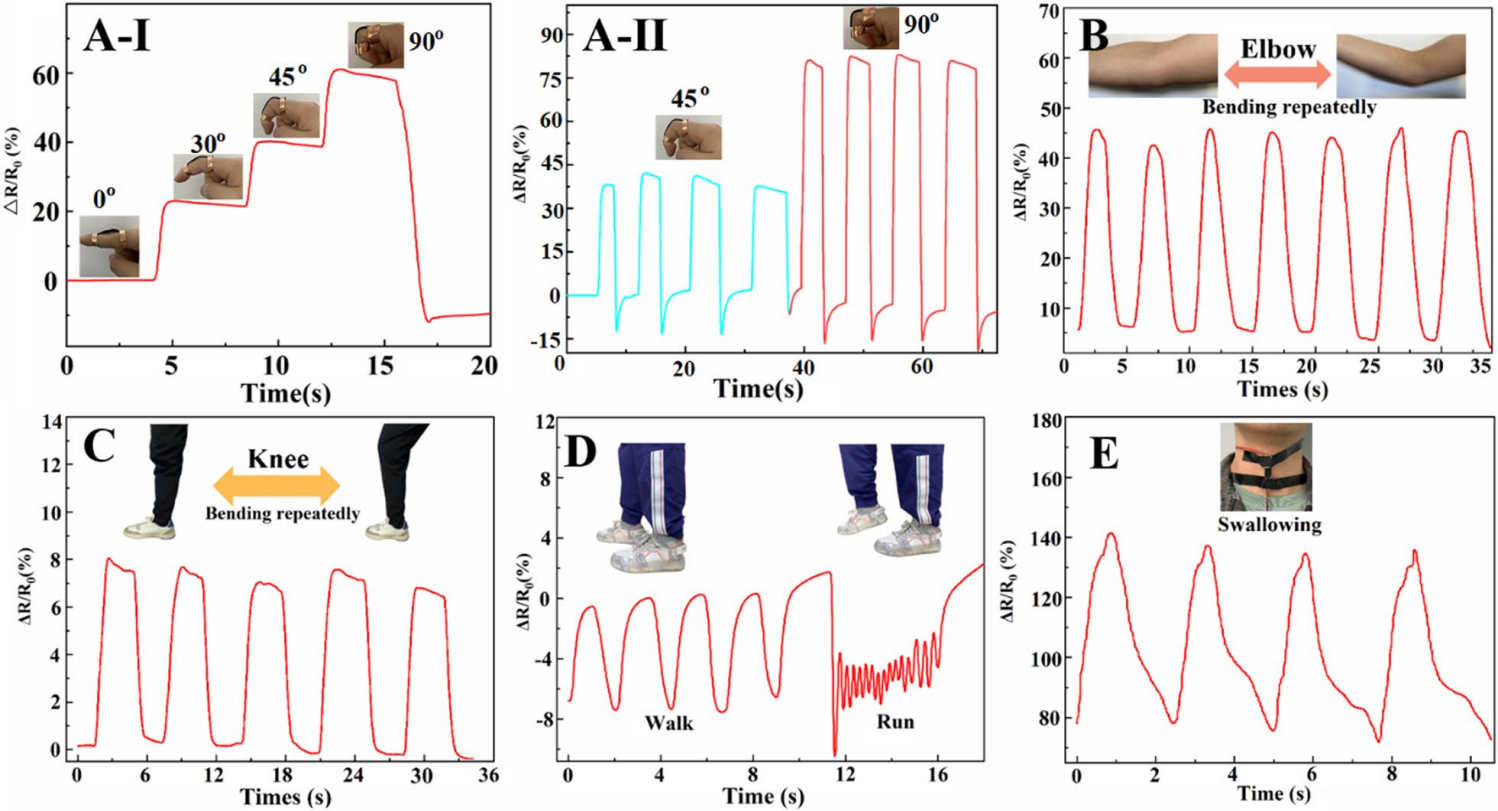
Monitoring human movement using the Hep_20_-PDA_0.8_-rGO-PAM hydrogel. Detection of finger bending angle variation (**A-I**), repeated finger bending at various angles (**A-II**), repeated arm flexion (**B**), repeated knee bending (**C**), walking and running activities (**D**), and repeated swallowing (**E**).

The reduction degrees of rGO were characterized by using UV–vis, Raman and FTIR spectra. The UV-vis spectrum of the GO suspension exhibited two characteristic peaks (Fig. 1A**-**I) at 230 nm and 300 nm, corresponding to the π-π* transitions of aromatic C=C bonds and n-π* transitions of the C=O bonds in GO, respectively. As the proportion of heparin increased, the absorption peak of Hep-rGO associated with the π-π* transition gradually red-shifted to a higher wavelength above 250 nm, indicating the mild reducing capacity of heparin. On the other hand, the absorption spectrum of PDA-rGO peaked at 261–279 nm, and the redshift became more significant with increasing PDA dosage (Fig. 1A**-**II), demonstrating the strong reducing capability of PDA towards GO. Comparing the peak values of Fig. 1A**-**III-1, 2 and 3, it can be observed that the longer wavelengths exhibited by Hep_20_-PDA_0.2_-rGO indicate a synergistic reduction of GO facilitated by the HEP-PDA complex. Furthermore, Hep_20_-PDA_0.8_-rGO shifted into a wavelength of around 283 nm, indicating a high degree of reduction of GO. The Raman spectra of GO displayed two prominent peaks at 1589 and 1357 cm^−1^ (Fig. 1B-1), corresponding to the G (the E_2g_ mode of sp^2^ carbon atoms) and D (the symmetry A_1g_ mode) bands, respectively. The intensity ratio of the D and G peaks (I_D_/I_G_) indicates the degree of disorder in graphene [24]. After the reduction process, the I_D_/I_G_ increased to varying degrees compared to GO (I_D_/I_G_= 0.92). Comparing the spectra of Fig. 1B-2, 3 and 4, Hep_20_-PDA_0.2_-rGO showed a higher I_D_/I_G_ ratio (I_D_/I_G_=1.06) compared to Hep_20_-rGO and PDA_0.2_-rGO, indicating synergistic reduction by the Hep-PDA complex. Hep_20_-PDA_0.8−_rGO exhibited the highest I_D_/I_G_ ratio, suggesting nearly complete reduction of GO when the PDA content in the Hep-PDA complex reached 0.8 wt%. The infrared spectra (Fig. 1C) of GO and rGO revealed characteristic peaks associated with oxygen functionalities in GO, such as O-H groups (3460 cm^−1^), C=O stretching (1740cm^−1^), and C–O stretching (1055 cm^−1^). With increasing Hep, PDA, and Hep-PDA contents, these characteristic peaks weakened or disappeared, indicating varying degrees of reduction in GO. The above analysis results show that Hep-PDA complex serve as an effective reducing agent to implement the controllable transformation of GO to rGO under moderate reaction conditions.

Figure 1D shows the thermogravimetric analysis (TGA) results. GO exhibits a rapid weight loss of 58.1% at 800 °C, mainly due to the removal of water molecules and the combustion of oxygen-containing functional groups [25]. In contrast, Hep_20_-PDA_0.8_-rGO demonstrates improved thermal stability, with a lower weight loss of only 27.8% between 100 and 800 °C. This indicates a significant reduction in oxygen-containing functional groups after the reduction of GO. Additionally, the stability of Hep_20_-PDA_0.8_-rGO in aqueous solution was evaluated over 3 months (Additional file 1: Fig. S1). Unlike Hep_20_-rGO and PDA_0.8−_rGO, Hep_20_-PDA_0.8_-rGO showed no signs of aggregation after three months. This enhanced stability is attributed to the synergistic effects of heparin and PDA. PDA aids in the absorption of heparin on the rGO surface and imparts a strong negative charge density (− 33 ± 0.60 mV), as confirmed by DLS (Additional file 1: Fig. S2) and shown in Additional file 1: Table S2. This negative charge prevents aggregation through electrostatic repulsion interactions, ensuring the stability of the Hep-PDA-rGO nanosheets. Figure 1E presents cross-sectional AFM images and corresponding height measurements of single-layered GO and rGO. The average thickness of the single-layer GO sheet was found to be 0.53 nm (Fig. 1E**-**I), consistent with previous literature [26]. After reduction and functionalization with heparin/PDA, the average thickness increased to 1.25 nm/2.19 nm, respectively. Notably, a further increase in average thickness to 2.57 nm was observed in Hep_20_-PDA_0.8_-rGO (Fig. 1E**-**IV), indicating successful immobilization of the Hep-PDA complex on the rGO surface.

### Preparation and mechanical characterization of Hep-PDA-rGO-PAM hydrogel

Flexible hydrogel sensors face periodic loading in practical applications, necessitating excellent mechanical properties to maintain stable sensing performance. The incorporation of rGO in the hydrogel plays a crucial role in achieving optimal properties. In this study, GO/rGO was added at a ratio of 1.0 wt% to the PAM network. The composition of each conductive hydrogel is shown in Additional file 1: Table S2. Functionalizing the rGO sheets with PDA or heparin led to improved compressive and tensile strength of the hydrogel (Fig. 2A**/**C-I, II-c, d relative to b). Particularly, the Hep_20_-PDA_0.2−_rGO-PAM hydrogel demonstrated higher compressive and tensile strength than Hep_20_-rGO-PAM and PDA_0.8_-rGO-PAM hydrogels, owing to the synergistic action of the Hep-PDA complex nanosheets (Fig. 2A**/**C-I, II-e relative to c, d). This enhancement can be attributed to the homogeneous dispersion of Hep_20_-PDA_0.2_-rGO within the polymer matrix, which enhances mechanical properties due to the nanomaterial’s large aspect ratio and interaction with the polymer chains [27]. However, increasing the PDA content resulted in a decrease in the mechanical strength of the Hep-PDA-rGO-PAM hydrogel (Fig. 2A**/**C-I, II-e, f, g) due to increased reduction of oxygen-containing groups on the GO nanosheets, weakening their interaction with the PAM network [21]. Cyclic compressive tests demonstrated that the compression performance of the Hep_20_-PDA_0.8_-rGO-PAM hydrogel remained stable after eight cycles (Fig. 2B). Additionally, the Hep_20_-PDA_0.8_-rGO-PAM hydrogel displayed high stretchability, with a maximum strain exceeding 350% (Fig. 2C-III-g) and a tensile strength of approximately 250 kPa (Fig. 2C**-**II-g), matching the elastic modulus of the skin. Thus, as a 2D nanomaterial, Hep-PDA-rGO effectively transfers loads and reinforces the mechanical properties of nanocomposite hydrogels, laying the foundation for the development of high-performance hydrogel sensors.

### Electrical conductivity and compression/strain sensing of hydrogels

rGO incorporation enhances the electrical conductivity of PAM hydrogel, which is crucial for sensing performance. In Fig. 3A-I, a Hep-PDA-rGO-PAM hydrogel-connected circuit with a lighted bulb demonstrates conductivity. Conductivity measurements using a two-probe method (Fig. 3A-II) show that the hydrogels exhibit different conductivities. Nyquist plots in Fig. 3B-I and corresponding conductivity results in Fig. 3B-II indicate that the hydrogel conductivity aligns with the rGO reduction degree. GO-PAM hydrogels have very low conductivity (0.16 S/m), but with increased PDA and Hep content, the degree of GO reduction and hydrogel conductivity increase. Notably, Hep-PDA-rGO composite hydrogel exhibits significantly enhanced conductivity compared to Hep-rGO and PDA-rGO hydrogels due to synergistic reduction by the Hep-PDA complex. Hep_20_-PDA_0.8_-rGO-PAM hydrogel, with only 1.0 wt% rGO, achieves a conductivity of 3.63 S/m, surpassing many reported ionic gel conductors [28, 29]. SEM micrographs reveal that the GO-PAM scaffold has smooth pore walls (Fig. 3C**-**I), while PDA-rGO nanosheets cluster on the scaffold (Fig. 3C**-**II). In contrast, Hep-PDA-rGO is uniformly scattered, forming an integrated electronic transmission pathway in the matrix (Fig. 3C**-**III). Such a big rise in conductivity is related to the higher content of sp^2^ carbon, reduced structural defects, and the integrity of the conductive network within the Hep_20_-PDA_0.8_-rGO-PAM hydrogel.

Hep_20_-PDA_0.8_-rGO-PAM hydrogel exhibits high sensitivity to compressive stress, as shown in Fig. 3D-I. The relative resistance change (ΔR/R_0_) decreases as the sample is compressed. This is attributed to the reduced interlayer spacing and increased contact area between graphene nanosheets under compression, leading to more efficient electron transmission and a decrease in the hydrogel’s resistance at a macroscopic level [30]. The hydrogel shows a pressure sensitivity of 0.464 KPa^−1^ at low pressure (< 0.2 MPa) and maintains a sensitivity of 0.037 KPa^−1^ at high pressure (around 1.0 MPa), covering a wide range of sensing working pressure. In Fig. 3D-II, it is shown that the △R/R_0_ value of the hydrogel exhibits a stable and incremental response to applied compression levels ranging from 5–50%. The repeatability of the compression-sensitive electrical performance is evaluated in Fig. 3D-III, where the hydrogel demonstrates relatively stable (a slight reduction due to water loss in the hydrogel material) and repeatable response signals with well-preserved amplitude even after undergoing 50 consecutive cycles of 20% compression. Furthermore, Fig. 3E-I illustrates the △R/R_0_ response of Hep_20_-PDA_0.8−_rGO-PAM hydrogel under applied tensile strain. The sensitivity of ΔR/R_0_ increases as the hydrogel is stretched. This can be attributed to the reduction in conductivity pathways for electrons on the graphene when the hydrogel is stretched, resulting in a decrease in electron mobility. The sensitivity value reaches 0.24 at 100% strain and 1.45 at 350% strain, indicating a wide feasible strain range of 0-350%. Figure 3E-II shows identical waveforms obtained in each cycle when maximum strains of 50%, 100%, and 150% are applied for 5 cycles, indicating the stability of the hydrogel’s response. Similarly, Fig. 3E-III demonstrates stable and repeatable strain response signals obtained when subjecting the hydrogel to a cyclic tensile strain of 100% for 50 consecutive cycles. Based on these findings, Hep_20_-PDA_0.8_-rGO-PAM hydrogel holds promise as a wearable sensor due to its stable resistive response to both compressive stress and tensile strain.

### PDA-HEP-rGO-PAM hydrogels for the epidermal sensor

Hep_20_-PDA_0.8_-rGO-PAM hydrogels were applied to different parts of the human body to evaluate their practical application as wearable sensors. Figure 4A**-**I shows that the ΔR/R_0_ increases as the finger is bent, and the electrical signals exhibit high repeatability and stability, as depicted in Fig. 4A**-**II. Similarly, when the hydrogel sensors are attached to joints such as elbows (Fig. 4B) and knees (Fig. 4C), they demonstrate sensitive and repeatable detection of joint bending. Moreover, the hydrogel sensors can accurately identify human walking and running by analyzing distinguishable resistance change frequencies and signal waveforms, as shown in Fig. 4D. Interestingly, even subtle human motions like swallowing can be easily detected by observing corresponding ΔR/R_0_ variations (Fig. 4E). The sensing sensitivity depends on the extent of changes in the conductive network during the strain process [31]. These findings demonstrate the potential of Hep_20_-PDA_0.8−_rGO-PAM hydrogel as a wearable sensor for monitoring various human activities in real time. Its ability to detect and distinguish movements in different body parts makes it a promising candidate for applications in healthcare and motion tracking.

### In vitro biocompatibility of hydrogels

**Fig. 5:**
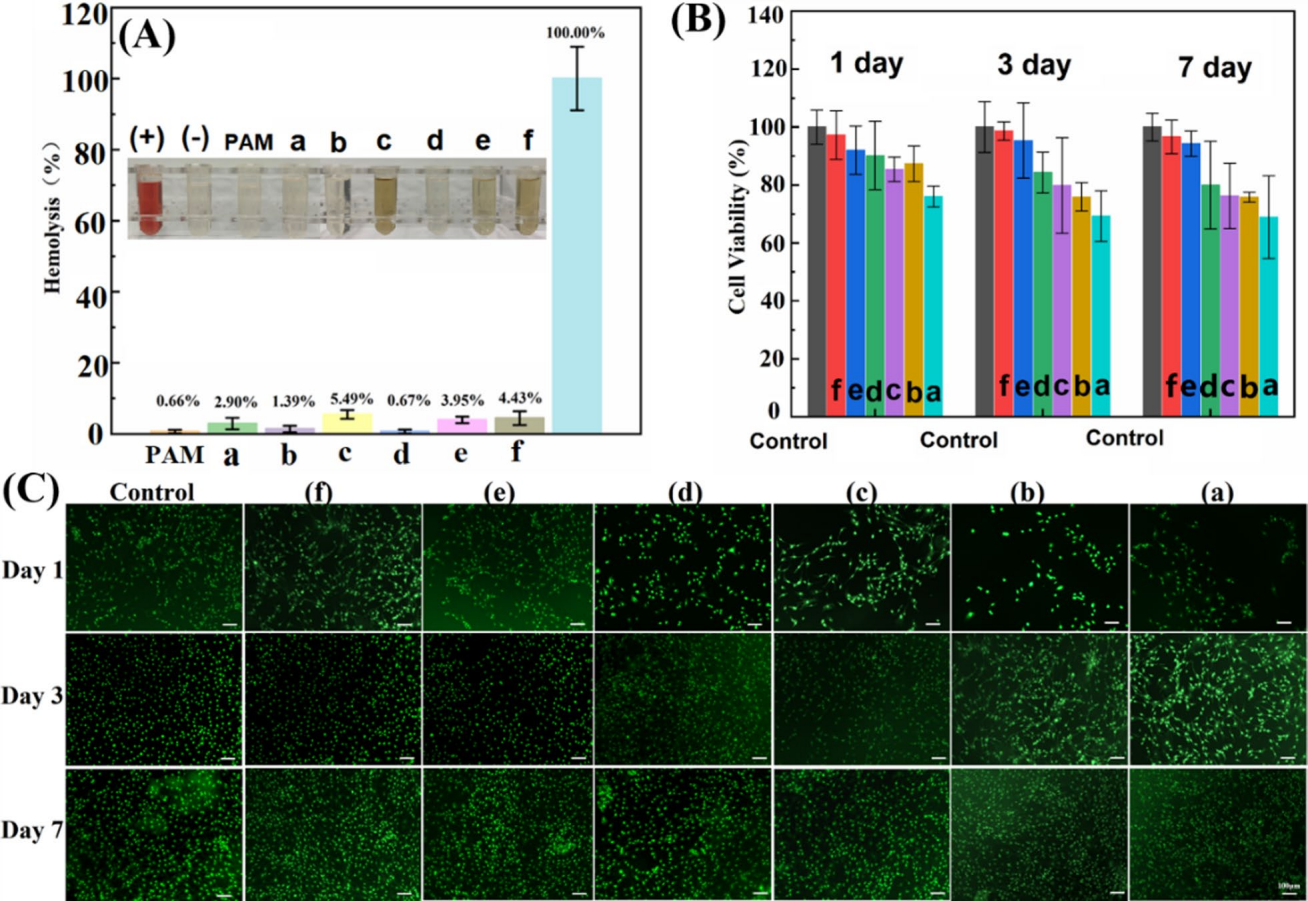
**A** Hemolysis ratios of RBCs with images of RBCs (inset) after the hydrogels incubated with RBCs for 2 h at 37 °C with shaking. The redness indicates that the RBCs were destroyed and hemoglobin was released. (+) represents positive control, and (−) represents negative control; Cell viability (**B**) and fluorescent staining (**C**) of 3T3 cells on hydrogel; GO-PAM (a), HEP_20_-rGO-PAM (b), PDA_0.8_-rGO-PAM (c), HEP_20_-PDA_0.2_-rGO-PAM (d), HEP_20_-PDA_0.4_-rGO-PAM (e), HEP_20_-PDA_0.8_-rGO-PAM (f)

Hemolysis ratio and viability studies on 3T3 cells were conducted to assess the biocompatibility of GO and rGO-based hydrogels. The hemolytic property of GO/rGO hydrogel is primarily influenced by the interactions between the incorporated nanosheets and blood components. In Fig. 5A, it can be observed that the GO-PAM hydrogel exhibited a higher hemolysis ratio (2.90%) compared to the pure PAM hydrogel (0.66%), suggesting that GO caused some disruption to the lipid bilayer of the blood cell membrane due to its surfactant and amphiphilic properties [32]. On the other hand, the Hep_20_-rGO-PAM hydrogel showed a lower hemolysis ratio (1.39%) due to the protective effect of heparin, which acts as an electric charge barrier and also alters the surface charge distribution of rGO [33]. However, the PDA_0.8_-rGO-PAM hydrogel displayed visible release of hemoglobin, with a high hemolysis rate of 5.49%, indicating a strong interaction between the PDA-adhered rGO and the blood cell membrane. This result aligns with the previous report, indicating that pristine graphene exhibited the highest hemolysis rate, followed by rGO and GO, in correlation with the degree of surface oxidation [34]. Interestingly, the Hep-PDA-rGO-PAM hydrogel exhibited significantly reduced hemolysis compared to the PDA_0.8_-rGO-PAM hydrogel, which can be attributed to the influence of the Hep-PDA complex on the surface modification of rGO. The hemolysis rate of the Hep_20_-PDA_0.8_-rGO-PAM hydrogel fell within the hemolytic standard (5%), indicating acceptable hemocompatibility of the hydrogel.

The cytotoxicity of GO and rGO-based hydrogels was assessed using the CCK-8 method against 3T3 cells, and the results are presented in Fig. 5B. Cell viability on the Hep-PDA-rGO-PAM hydrogel was consistently high, exceeding 90% throughout the experiment. In contrast, other hydrogel groups showed a decrease in cell viability over time, with the GO-PAM hydrogel group reaching as low as 70%. Particularly, the Hep_20_-PDA_0.8−_rGO-PAM hydrogel demonstrated excellent cell compatibility with a cell survival rate of 97%. These findings indicate that cells had a higher proliferation rate on the Hep-PDA-rGO-PAM hydrogel, and the density and growth of cells were higher on hydrogels with a higher PDA content. AO/EB staining confirmed the cytotoxicity results, showing increased cell density over time, particularly in the Hep_20_-PDA_0.8_-rGO-PAM (Fig. 5C).

The results indicate that GO-PAM induces hemolysis and cytotoxicity due to its surface properties and interaction with the cell membrane. In contrast, the presence of PDA reduces electrostatic repulsion between rGO and the cell membrane, leading to its partitioning into the lipid bilayer. However, when PDA forms a complex with heparin, it enhances adhesion of the heparin layer to the rGO surface, resulting in reduced hemolysis. Regarding cytotoxicity, cells cultured with PDA_0.8_-rGO-PAM and Hep_20_-PDA_0.8_-rGO-PAM hydrogels show improved spreading morphology on the first day, likely due to PDA facilitating cell attachment [35]. Moreover, the limited aqueous stability of rGO causes sedimentation and compact aggregate formation, limiting nutrient availability to cells [36]. Hep_20_-PDA_0.8_-rGO-PAM hydrogel exhibits low cytotoxicity, possibly due to the higher aqueous stability of Hep_20_-PDA_0.8_-rGO nanosheets. It is worth noting that the stability of different GO/rGO samples correlates with their corresponding hydrogel cell viability.

### In vitro reactive oxygen species (ROS) scavenging activities of hydrogels

During an injury or implantation, the body responds rapidly by accumulating cells and biomolecules to initiate the healing process at the affected site. However, this response also triggers the generation of ROS and free radicals from neighboring tissues and immune reactions. While ROS plays a crucial role as a signaling molecule in regulating physiological processes, excessive ROS production due to inflammation at the implant site can exacerbate cellular damage and compromise the stability of sensor devices [37]. Furthermore, the hindrance to diabetic wound healing arises from an overabundance of inflammatory cytokines, ROS, and cellular dysfunction, compounded by the intrinsic antioxidative defense system’s inability to rectify these imbalances. Consequently, the antioxidant properties that effectively counteract ROS oxidation reactions become paramount not only for implanted flexible sensors but also for chronic wound dressing. In this study, we investigated the ROS scavenging properties of hydrogels, assessing their ability to inhibit hydroxyl radicals (OH·) and DPPH radicals, highlighting their potential as protective and treatment components within the realms of implantable sensors and wound dressing.

**Fig. 6:**
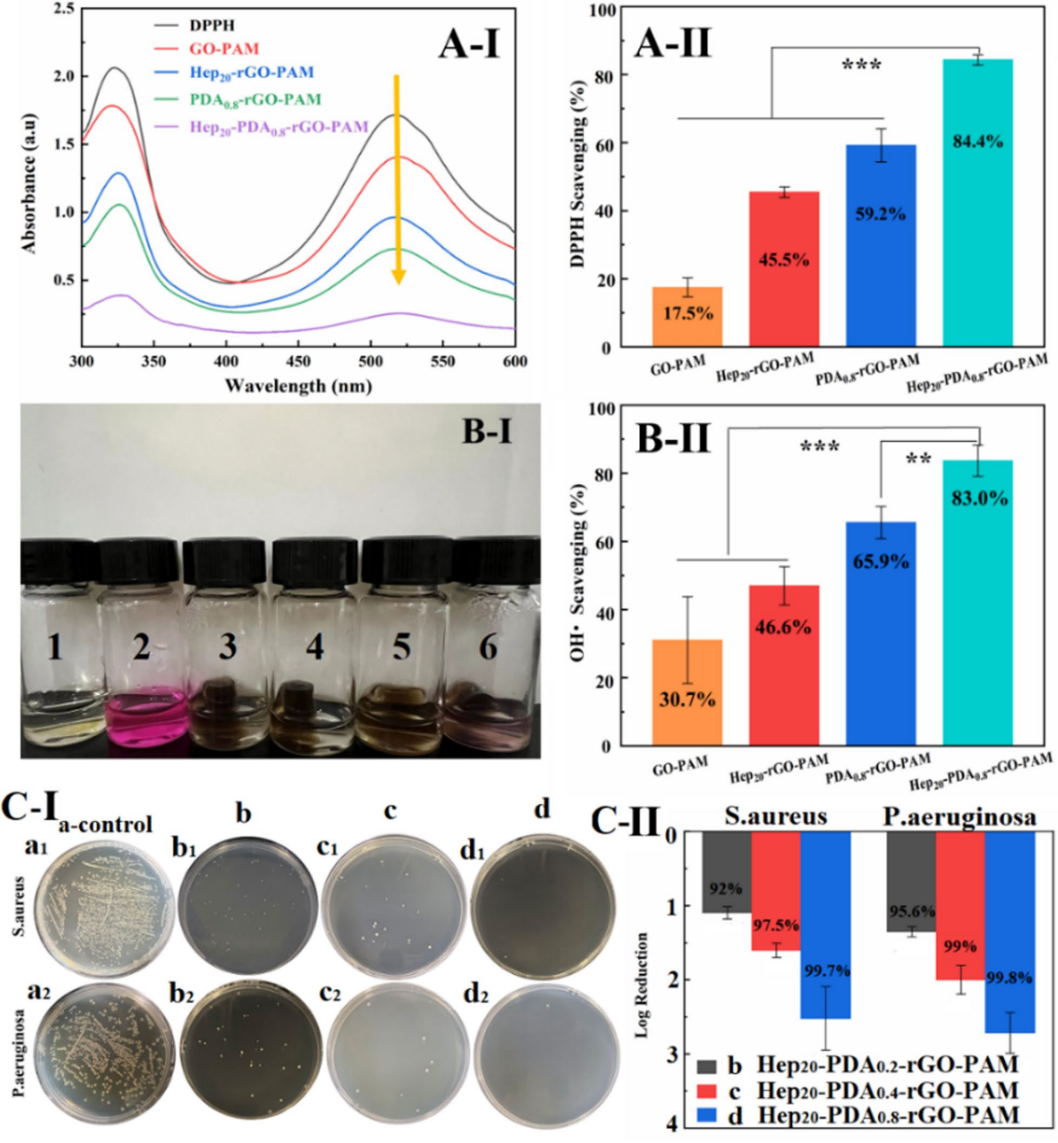
ROS scavenging property of hydrogels against Hydroxyl radicals and DPPH radicals. (**A-I**) UV − vis spectra of the DPPH reaction with different scaffolds after 120 min, (**A-II**) DPPH scavenging efficiency of the scaffolds; (**B-I**) The OH• scavenging activities of samples were demonstrated by the color change (1: blank, 2: control, 3: GO-PAM, 4: Hep_20_-rGO-PAM, 5: PDA_0.8_-rGO-PAM, 6: Hep_20_-PDA_0.8_-rGO-PAM group), (**B-II**) OH• scavenging efficiency of the scaffolds; (**C-I**) Growth conditions of *S. aureus* (a_1_-d_1_) and *P. aeruginosa* (a_2_-d_2_) on the blank culture substrate (a_1_, a_2_), Hep_20_-PDA_0.2_-rGO-PAM (b_1_, b_2_), Hep_20_-PDA_0.4_-rGO-PAM (c_1_, c_2_) and Hep_20_-PDA_0.8_-rGO-PAM (d_1_, d_2_) hydrogel; (**C-II**) Antibacterial rate calculated by log reduction of bacteria (CFU) on contact with the three hydrogels according to the spread plate results (n = 3)

In the free DPPH radicals scavenging assay, the intensity of the DPPH radical was measured after 60 minutes of incubation with the hydrogel by monitoring the characteristic UV-Vis absorption peak of DPPH radicals at 517 nm. As depicted in Fig. 6A**-**I, the peak value decreased from 1.71 to 0.25 in the presence of GO/rGO hydrogels. The GO-PAM hydrogel exhibited a moderate scavenging capacity against DPPH (17.5%), while the scavenging efficiency of Hep_20_-rGO-PAM and PDA_0.8_-rGO-PAM hydrogels was enhanced to 45.5% and 59.2%, respectively (Fig. 6A**-**II). This enhancement suggests the crucial role of reductive hydroxyl groups and catechol groups present on rGO as electron donors in the scavenging process [38]. Importantly, the introduction of the Hep-PDA complex significantly enhanced the DPPH scavenging capacity of rGO hydrogels to 84.4%, attributed to the synergistic inhibition of heparin and PDA (Fig. 6A**-**II). In the OH· scavenging assay, the Fenton reaction was employed to generate hydroxyl radicals. Hydrogen peroxide was reduced to OH· by ferrous ions, and the strong oxidizing ability of OH· led to the oxidation and discoloration of the Safranin-O dye [39]. Thus, the darker the color of the reaction solution, the stronger the hydrogel’s scavenging ability towards OH·. It was observed that the solution color of the GO-PAM hydrogel group was the lightest, and the color gradually darkened with the introduction of Hep, PDA, and Hep-PDA, as shown in Fig. 6B**-**I. The Hep_20_-PDA_0.8_-rGO-PAM hydrogel exhibited the highest scavenging capacity against OH· at 83% (Fig. 6B**-**II). This can be attributed to the hydroxyl groups and phenolic compounds on the Hep-PDA complex, which bind to OH· as electron donors and inhibit OH· generation.

### In vitro antibacterial activities of hydrogels

Chronic wound infections are highly prevalent among diabetic patients, stemming from compromised immunity due to high blood sugar levels, impaired circulation, and neuropathy, collectively resulting in increased susceptibility and delayed healing. Conventional conductive hydrogels have a drawback of microbial absorption, which can lead to inflammation, wound infections, and immune responses, posing a risk to human health. Graphene materials have emerged as potential antibacterial agents due to their low toxicity and broad-spectrum activity [40]. Our previous research has demonstrated that heparin enhances the antimicrobial activity of PDA through the bactericidal effects of cations and ROS [23, 41]. To evaluate the antibacterial activity of the hydrogels, two representative clinical pathogens, namely *S. aureus* and *P. aeruginosa*, were cultured on the surface of the hydrogels at a concentration of ~ 10^6^ CFU/cm^2^ for 4 hours. The antibacterial activity was assessed using the plate colony counting method with co-cultured bacterial suspensions. The blank culture substrate served as the control (Fig. 6C**-**I-a). As depicted in Fig. 6C**-**I-b, c, d, Hep-PDA-rGO-PAM hydrogel exhibited substantial reduction in bacterial colonization, surpassing 90% eradication for both pathogens. Moreover, an elevated PDA content led to even lower bacterial counts. In particular, the Hep_20_-PDA_0.8_-rGO-PAM hydrogel achieved nearly complete elimination of *S. aureus* (99.7% kill-log reduction at 2.5) and *P. aeruginosa* (99.8% kill-log reduction at 2.65), indicating its robust antibacterial efficacy (Fig. 6C**-**II). This synergistic effect between the Hep-PDA complex and rGO nanosheets contributes to the antibacterial properties of the conductive hydrogel by disrupting microbial membranes.

### In vivo wound healing effects

**Fig. 7:**
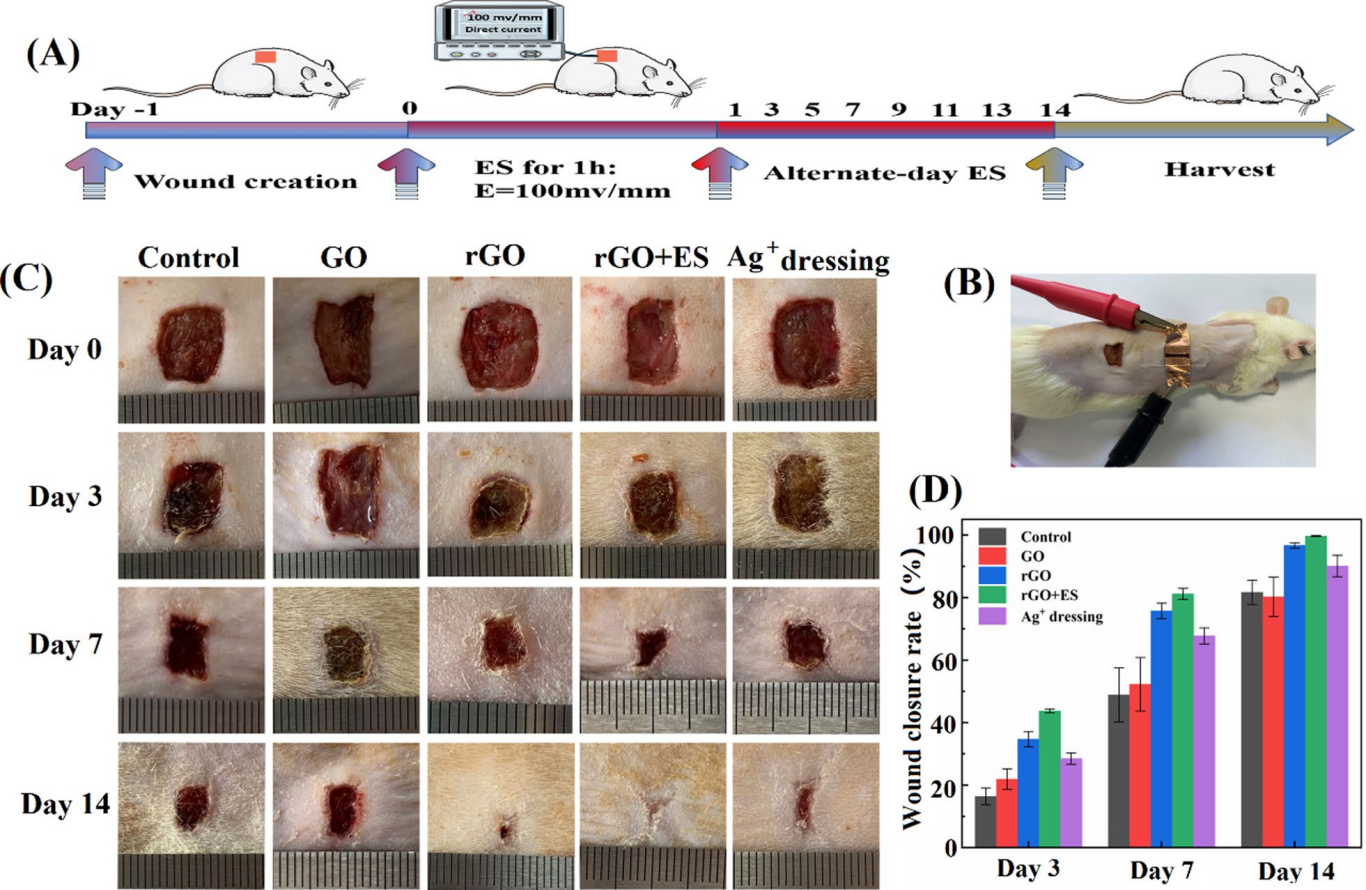
Schematic representation of in vivo wound healing experiment process (**A**), Digital image of electrical stimulation treatment process (**B**), Photographs of wounds on day 0, 3, 7, 14 days from the different treatment (**C**), Wound closure rate as a function of time for each treated group (**D**)

The Hep-PDA-rGO-PAM hydrogel has great potential as an advanced dressing for electrotherapy (ES) in the treatment of chronic wounds due to its conductivity and multi-functionalities. To evaluate its wound healing performance, the Hep_20_-PDA_0.8_-rGO-PAM hydrogel was compared to the GO-PAM hydrogel (referring to rGO and GO hydrogel in the following context) in a full thickness skin defect model on type II diabetes mellitus Sprague-Dawley (SD) rats. In the study, an external ES powered by a DC supply was applied to the rGO hydrogel for one hour on alternate days in the rGO + ES group, to investigate the combined effects of rGO hydrogel dressings with ES (Fig. 7A, B). The control group received no treatment except saline solution, while the Ag^+^ dressing group was treated with a commercial Ag^+^ dressing (AQUACEL®). All wounds were inoculated with *S. aureus* to induce infection. The macroscopic appearances of the wounds with different treatments on day 0, 3, 7, and 14 were recorded (Fig. 7C). The wound area decreased over time in all groups albeit at different rates: rGO + ES group < rGO hydrogel group < Ag^+^ group < GO hydrogel group < control group. Particularly, the wound treated with ES through the rGO hydrogel showed the smallest wound area among all the groups at any time point, achieving almost complete (99.7%) wound closure after 14 days (Fig. 7D). Furthermore, a significant reduction in wound size was observed in the rGO group without ES, compared to the GO hydrogel group and Ag^+^ dressing group.

**Fig. 8:**
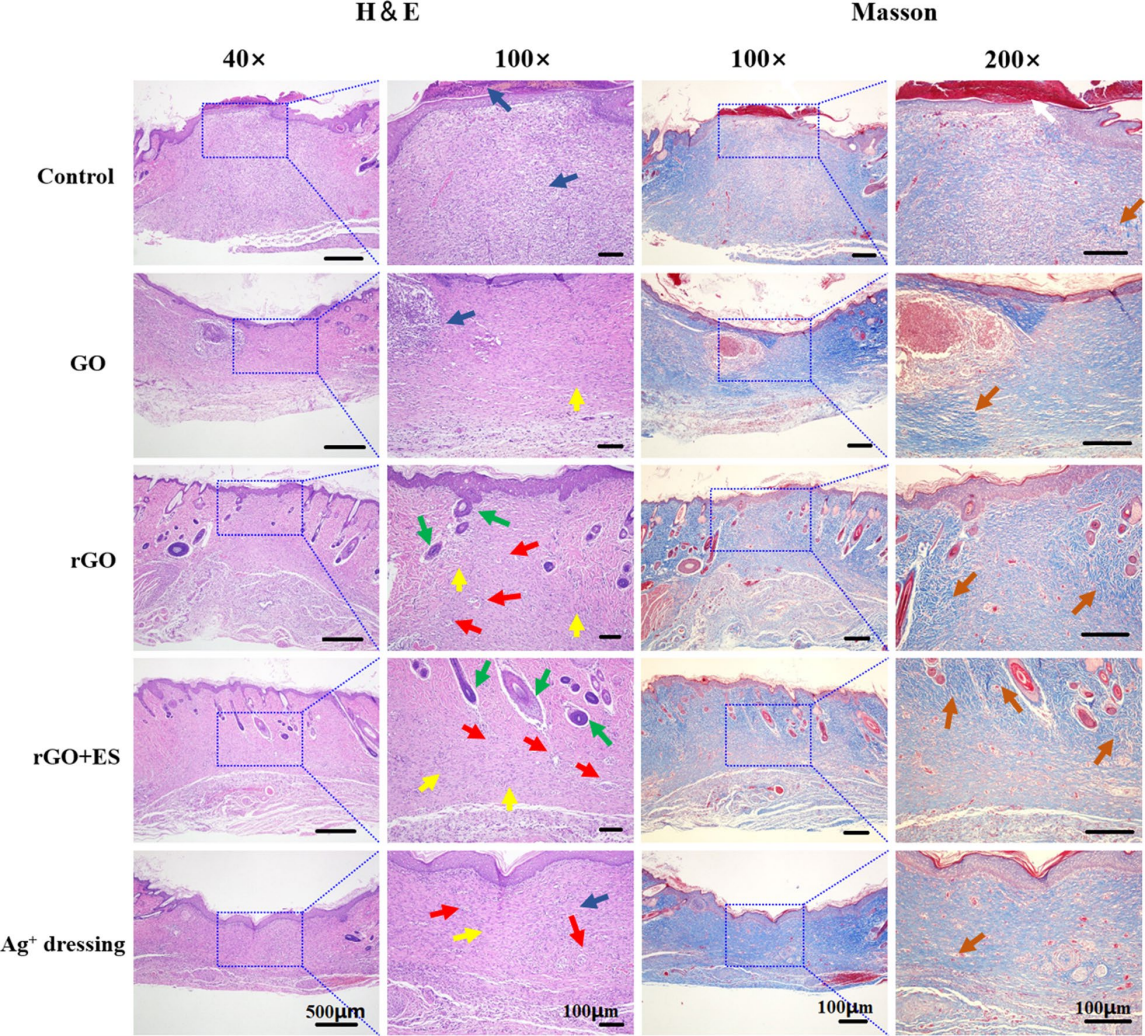
Hematoxylin & eosin and Masson staining of the sections of the regenerated tissues at the wound site on day 14 for the different treated groups. Scab (white arrow), blood vessels (red arrow), fibroblasts (yellow arrow), neutrophils (blue arrow), hair follicles (green arrow), and collagen deposition (orange arrow)

Histological analysis of tissue sections from day 14 wounds was performed using H&E and Masson staining. The analysis revealed distinct variations among the different treatment groups (Fig. 8). The control group displayed incomplete wound closure, characterized by the presence of a scab and a high number of blood cells and inflammatory cells. In the Ag^+^ film dressing group, basic formation of epithelium and dermis was observed; however, the dermis was still undergoing repair with mild inflammation and the absence of hair follicles. The GO hydrogel group exhibited incomplete epithelium and dermis, with loosely distributed dermal tissue and a significant infiltration of neutrophils. In contrast, the rGO hydrogel demonstrated regular formation of epithelium and connective tissue, accompanied by an increased density of fibroblasts, a reduced presence of neutrophils and new blood vessels and hair follicles formation. In general, excessive inflammation can delay the healing, while the rGO group, due to its antibacterial and antioxidative properties, maintained an appropriate inflammatory environment for promoting wound healing. Remarkably, the rGO + ES group exhibited the most favorable wound healing outcomes, including abundant mature hair follicles and neoangiogenesis, as well as well-proliferated fibroblasts, surpassing the results of all other groups. Fibroblasts play a key role in producing collagen through their rapid proliferation [42]. Collagen, as the primary constituent of the extracellular matrix, plays a crucial role in the process of wound healing. After a 14-day period, the wounds treated with rGO hydrogel and ES exhibited the highest density of collagen (represented by the blue area) and a more organized fibrous structure, facilitating connective tissue remodeling compared to the other groups.

**Fig. 9:**
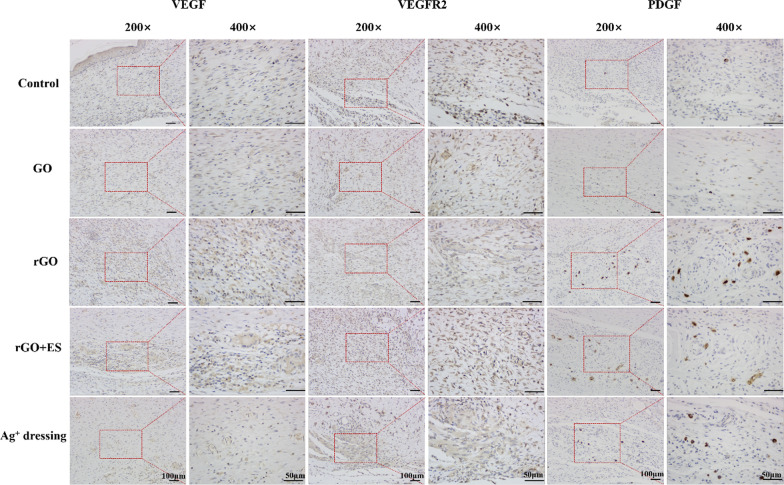
Immunohistochemical staining of VEGF, PDGF and VEGFR2 growth factors at the wound sites on day 14 after different treatments.

Angiogenesis, the formation of new blood vessels, is vital for wound repair as it contributes to the development of granulation tissue and supplies nutrients and oxygen to growing tissues [43]. Several growth factors, including VEGF, PDGF, and VEGFR2, are involved in angiogenesis during wound healing. VEGF enhances vascular permeability and attracts endothelial cells, initiating angiogenesis. PDGF stimulates the proliferation of smooth muscle cells (SMCs) to enhance vascular stability. VEGFR2, as a receptor for VEGF, binds to VEGF and activates intracellular signaling pathways, promoting the growth, proliferation, and maturation of endothelial cells and facilitating neovascularization [44]. Therefore, immunohistochemical analysis of VEGF, PDGF and VEGFR2 can be used to evaluate angiogenesis during the wound healing process. As shown in Fig. 9, the expression levels of VEGF, VEGFR2, and PDGF (indicated by the brownish-yellow staining) in the rGO groups were obviously higher than in the other groups. Specifically, when exposed to ES, the expression of the VEGFR2 in the rGO + ES group was notably higher compared to both the Ag^+^ dressing and rGO hydrogel treated groups. This suggests that electrical stimulation via rGO hydrogel can promote angiogenesis and improve the healing rate of wound by upregulating the expression levels of VEGF, VEGFR2, and PDGF. All above findings suggest that the electroactive rGO hydrogel alone can enhance skin wound healing due to its intrinsic properties, such as antibacterial and antioxidative effects, and when combined with ES, it exhibits accelerated therapeutic effects, indicating a synergistic role between the rGO hydrogel and ES.

## Conclusion

In this study, the Hep-PDA complex was employed to enhance the stability, reducibility, and biocompatibility of rGO through reduction modification of GO. The resulting Hep-PDA-rGO nanosheets were incorporated into a PAM hydrogel matrix, resulting in a multifunctional rGO-based conductive hydrogel. The conductive hydrogel possesses a range of desirable properties, including electroactivity, antioxidant activity, antibacterial activity, and excellent sensing performance, making it suitable for use as an epidermal sensor and also as an active diabetic wound dressing. The hydrogel can be directly applied to biological surfaces, allowing for real-time monitoring of both large-scale and subtle movements of the human body. Specifically, the Hep_20_-PDA_0.8_-rGO-PAM hydrogel effectively accelerates wound healing by managing wound infection, sustaining an appropriate inflammatory milieu, promoting angiogenesis, and aiding in collagen deposition. This work presents a novel approach for the design and construction of multifunctional hydrogels for diverse biomedical applications, encompassing diagnostics and therapies.

### Supplementary Information

**Additional file 1: Table S1. **Content of various components in conductive hydrogel. **Figure S1.** Dispersion stability of different component nanosheets in water. **Figure S2.** Particle size distribution and potential of GO nanosheets in water. **Table S2.** Particle size and potential of rGO nanosheets at different ratios.

## Data Availability

The raw/processed data required to reproduce these findings will be made available on request.
