# Supplementary material for: Multi-functional conductive hydrogels based on heparin–polydopamine complex reduced graphene oxide for epidermal sensing and chronic wound healing
Source: J Nanobiotechnology. 2023 Sep 23;21:343. doi: 10.1186/s12951-023-02113-9 (PMC10517544; doi:10.1186/s12951-023-02113-9)
Supplement: Supplementary file 1 — Additional file 1: Table S1. Content of various components in conductive hydrogel. Figure S1. Dispersion stability of different component nanosheets in water. Figure S2. Particle size distribution and potential of GO nanosheets in water. Table S2. Particle size and potential of rGO nanosheets at different ratios. [file 12951_2023_2113_MOESM1_ESM.docx]

Additional Information

**Multi-Functional Conductive Hydrogels Based on Heparin-Polydopamine Complex Reduced Graphene Oxide for Epidermal Sensing and Chronic Wound Healing**

Yiyong Dou^#^, Yuwei Zhang^#^, Shuo Zhang, Shuo Ma and Hong Zhang*

Key Laboratory of Biomaterials of Guangdong Higher Education Institutes, Department of Biomedical Engineering, Jinan University, Guangzhou 510632, China

*Corresponding author. E-mail address: zhanghong@jnu.edu.cn (H. Zhang)

**Table of contents**

**Experimental Section:**

1. Preparation of Conductive Nanosheets. P.2

2. Preparation of Conductive Hydrogel. P.2-3

3. Characterization of GO and rGO Nanosheets. P.3-4

4. Characterization of Conductive Hydrogel. P.4-5

5. Characterization of Sensing Performance of Conductive Hydrogel. P.5

6. Real-Time Monitoring of Human Motion Signals. P.6

7. Evaluation of Biocompatibility. P.6-7

8. Antibacterial Performance Evaluation. P.7-8

9. Antioxidant Performance Evaluation. P.8-9

10. Evaluation of Conductive Hydrogel in Promoting Chronic Wound Healing in Diabetic Rats. P.9-10

**Results:**

Table S1. Content of various components in conductive hydrogel P.11

Figure S1: Dispersion stability of different component nanosheets in water. P.12

Figure S2: Particle size distribution and potential of GO nanosheets in water. P.13

Table S2: Particle size and potential of rGO nanosheets at different ratios. P.14

**1. Preparation of Conductive Nanosheets:**

(1) Preparation of Hep-rGO Nanosheets: Firstly, heparin (Hep) is dissolved in deionized water at room temperature. After thorough stirring and complete dissolution, a dispersion of graphene oxide (GO-single layer) is added. The reaction is vigorously stirred for 20 minutes to achieve reduction.

(2) Preparation of PDA-rGO Nanosheets: Firstly, dopamine (DA) is dissolved in deionized water at room temperature. 1M NaOH is added to adjust the pH to 11. Then, under stirring, DA undergoes self-polymerization and oxidation in air for 20 minutes. Subsequently, a dispersion of GO is added, and the reaction is vigorously stirred for 10 minutes to achieve reduction.

(3) Preparation of Hep-PDA-rGO Nanosheets: Firstly, dopamine (DA) is dissolved in deionized water at room temperature. 1M NaOH is added to adjust the pH to 11. Then, under stirring, DA undergoes self-polymerization and oxidation in air for 20 minutes. Subsequently, a dispersion of Hep-rGO is added, and the reaction is vigorously stirred for 10 minutes to achieve reduction.

**2. Preparation of Conductive Hydrogel:**

Building upon the steps outlined in 1-(1), (2), and (3), acrylamide (AM), ammonium persulfate (APS), N,N'-methylenebisacrylamide (MBA), and N,N,N',N'-tetramethylethylenediamine (TMEDA) are added. The reaction is stirred under ice bath conditions. The prepolymer solution is then transferred to a mold and allowed to solidify, resulting in the formation of Hep-rGO-PAM, PDA-rGO-PAM, and Hep-PDA-rGO-PAM conductive hydrogels. The composition of each conductive hydrogel is presented in Table S1.

**3. Characterization of GO and rGO Nanosheets:**

(1) Particle Size and Zeta Potential Measurement: The samples are placed in an electrode cell, and the particle size distribution and zeta potential are measured using a laser particle size analyzer (Malvern Instruments Ltd, UK) at room temperature. Each sample is measured three times for accuracy.

(2) Solution Stability Analysis: Different nanosheets dispersed in water at a concentration of 100 mg/L are observed for stability over a period of three months.

(3) Thermogravimetric Analysis: The dispersion of rGO is freeze-dried and ground into a powder. The powder is subjected to thermogravimetric analysis (TGA, Mettler Toledo, Switzerland) under a nitrogen atmosphere, heating from room temperature to 800 °C at a rate of 10 °C/min. The obtained data is analyzed and plotted.

(4) UV-Visible Spectroscopy: UV-visible spectra of GO and rGO are obtained using a visible spectrophotometer (UV-2550) by scanning the absorbance in the range of 200-400 nm. The data is analyzed and plotted.

(5) Infrared Spectroscopy: The dispersion of rGO is freeze-dried and ground into a powder. Fourier-transform infrared spectroscopy (FT-IR, VERTEX70, Germany) is used to scan the powder in the range of 4000 cm-1 to 500 cm-1. The obtained data is analyzed and plotted.

(6) Raman Spectroscopy: The dispersions of GO and rGO are placed on glass slides and dried. Raman spectroscopy (Raman, HORIBA Jobin Yvon S.A.S, France) is performed to dynamically scan the samples in the range of 800 cm-1 to 2500 cm-1. The obtained data is analyzed and plotted.

**4. Characterization of Conductive Hydrogel:**

(1) Microscopic Morphology of PDA-Hep-rGO Conductive Hydrogel: Different compositions of conductive hydrogel are freeze-dried in a freeze dryer. After removing the moisture, the cross-section of the hydrogel samples is gold-sputtered and observed using a field emission scanning electron microscope (Ultra-55) to examine their microscopic morphology.

(2) Mechanical Properties of PDA-Hep-rGO Conductive Hydrogel: Compression Testing of Hydrogel: Cylindrical hydrogel samples with a radius of 5 mm and a thickness of 6-7 mm are placed on a universal testing machine. Compression tests are conducted at a deformation of 60% with a speed of 5 mm/min. Each sample is tested in triplicate. Tensile Testing of Hydrogel: Strip-shaped hydrogel samples with a length of 20-30 mm, width of 10 mm, and thickness of 1-2 mm are placed on a universal testing machine. Tensile tests are conducted at a speed of 20 mm/min until the samples fracture. Each sample is tested in triplicate.

(3) Electrical Conductivity of PDA-Hep-rGO Conductive Hydrogel: The electrical conductivity of the conductive hydrogel is measured using the two-electrode method. The hydrogel is sandwiched between two testing electrodes, forming a sandwich-like structure, and connected to an electrochemical workstation (Chi660c, Shanghai Huachen, China). The conductivity of the hydrogel is measured using impedance spectroscopy in the frequency range of 1 Hz to 100,000 Hz. The conductivity (σ) is calculated using formula 1.1, where d is the thickness of the hydrogel, R is the intercept on the x-axis of the Nyquist plot, and S is the contact area of the testing electrodes.

$\boldsymbol{\sigma=}\frac{\mathbf{d}}{\mathbf{R*S}}$ **(Formula 4.1)**

**5. Characterization of Sensing Performance of Conductive Hydrogel:**

(1) Compression Sensing Performance Testing: Cylindrical hydrogel samples with a radius of 5 mm and a thickness of 6-7 mm are placed on a universal testing machine. The universal testing machine is connected to an electrochemical workstation via wires. The electrochemical workstation is set to the i-t mode with a voltage of 1.0V. Real-time current and compression data are recorded. The compression sensitivity factor (S, Kpa^-1^) is calculated using formula 5.1.

$\boldsymbol{S}\boldsymbol{=\delta}\mathbf{（}\frac{\mathbf{R-Ro}}{\mathbf{Ro}}\mathbf{）}\boldsymbol{/ \delta}\mathbf{（}\mathbf{P}\mathbf{）}$**(Formula 5.1)**

Where R is the real-time resistance of the hydrogel sample, Ro is the initial resistance of the hydrogel sample at rest, and P is the real-time applied stress on the hydrogel sample during compression.

(2) Tensile Sensing Performance Testing: Strip-shaped hydrogel samples with a length of 20-30 mm, width of 10 mm, and thickness of 1-2 mm are placed on a universal testing machine. The universal testing machine is connected to an electrochemical workstation via wires. The electrochemical workstation is set to the i-t mode with a voltage of 1.0V. Real-time current and strain data are recorded. The tensile sensitivity factor (SF) is calculated using formula 5.2.

$\boldsymbol{SF}\mathbf{=}\frac{\mathbf{R-Ro}}{\mathbf{Ro}}\mathbf{/Ɛ}$ **(Formula 5.2)**

Where R is the real-time resistance of the hydrogel sample, Ro is the initial resistance of the hydrogel sample at rest, and Ɛ is the real-time applied strain on the hydrogel sample during tensile deformation.

**6. Real-Time Monitoring of Human Motion Signals:**

To demonstrate the feasibility of using the conductive hydrogel as a flexible sensor for real-time monitoring of human motion, the hydrogel samples are fixed to the monitoring sites on the human body, such as fingers, knees, and throat, using conductive adhesive tape. The hydrogel samples are then connected to an electrochemical workstation. The electrochemical workstation is set to the i-t mode with a voltage of 1.0V. Real-time changes in resistance during various human motions are monitored and recorded.

**7. Evaluation of Biocompatibility:**

(1) Hemocompatibility Evaluation: To evaluate blood compatibility, sodium citrate-anticoagulated rabbit blood is centrifuged at 5000 rpm for 5 minutes. The lower precipitate is collected and mixed with PBS to prepare a red blood cell (RBC) solution. The hydrogel samples are washed and sterilized before the experiment. Each hydrogel sample is soaked in a mixture of RBC solution and PBS and incubated in a constant temperature incubator at 37°C for 2 hours. A mixture of RBC and deionized water is used as the positive control group, and a mixture of RBC and PBS is used as the negative control group. After 2 hours, the solution is centrifuged at 5000 rpm for 5 minutes, and the absorbance of the supernatant at 540 nm is measured using an enzyme-linked immunosorbent assay (ELISA) reader. The hemolysis rate (HR) of the hydrogel is calculated using formula 7.1.

$\boldsymbol{HR}\mathbf{（}\boldsymbol{\%}\mathbf{）}\mathbf{=}\frac{\mathbf{OD}\mathbf{（}\mathbf{exp}\mathbf{）}\mathbf{-OD}\mathbf{（}\mathbf{N}\mathbf{egatiive}\mathbf{）}}{\mathbf{OD}\mathbf{（}\mathbf{P}\mathbf{ositive}\mathbf{）}\mathbf{-OD}\mathbf{（}\mathbf{N}\mathbf{egative}\mathbf{）}}\boldsymbol{100\%}$**(Formula 7.1)**

(2) Cytotoxicity Evaluation:

Extraction of Hydrogel Extract: The hydrogel is freeze-dried, sterilized using UV and ethanol, and washed with PBS to remove surface ethanol. The sterilized hydrogel samples are placed in a solution of 0.1 g/mL DMEM culture medium and incubated in a cell culture incubator at 37°C for 24 hours. The hydrogel extract is collected by filtering through a 0.22 μm filter and supplemented with fetal bovine serum and penicillin-streptomycin solution. This yields the hydrogel extract.

CCK-8 Assay for Cell Viability: Mouse fibroblast cells (3T3) with a cell density of 5000 cells/well are seeded in a 96-well plate and incubated at 37°C for 6 hours. The culture medium is removed, and 100 μL of the hydrogel extract is added to each well. The plate is then incubated in the cell culture incubator for 1 day, 3 days, and 5 days. The control group receives DMEM and cell suspension, while the blank group receives only DMEM culture medium. Each well is tested in 6 replicates. At specific time points, the culture medium is removed, and each well is washed three times with PBS (pH=7.4). Then, 100 μL of DMEM solution containing 10% (v/v) CCK-8 is added to each well and incubated in a CO_2_ incubator for 2-4 hours. The absorbance at 450 nm is measured using an ELISA reader, and the data are plotted.

Cell Fluorescent Staining: Mouse fibroblast cells (3T3) with a cell density of 5000 cells/well are co-cultured with the hydrogel extract for 1 day, 3 days, and 5 days. After incubation, the culture medium is removed, and the wells are washed three times with PBS (pH=7.4). The cells are then stained with a prepared acridine orange/ethidium bromide (AO/EB) fluorescent staining solution and incubated at 37°C for 10-20 minutes. After staining, the cells are washed three times with PBS and observed using a fluorescence inverted microscope.

**8. Antibacterial Performance Evaluation:**

Plate Count Method: The hydrogel samples with a diameter of 6 mm and a height of 8-9 mm are sterilized using UV light. A 10 μL suspension of S. aureus and P. aeruginosa bacteria with a concentration of 10^6 cfu/mL is placed on the top of the hydrogel. The samples are co-cultured with the bacteria in a bacterial incubator at 37°C for 2-4 hours. After incubation, 1 mL of PBS is added to the top of the hydrogel, and ultrasonication is performed to remove the bacteria. Then, 100 μL of the bacterial solution obtained after ultrasonication is evenly spread on an agar plate. The agar plate is incubated at 37°C for 15-18 hours. After incubation, the agar plate is taken out, photographed, and the colonies are counted. The antibacterial rate (R) is calculated using formula 8.1.

**R（%）=[1-10^（-lg**$\frac{\boldsymbol{N}_{\mathbf{0}}}{\boldsymbol{N}}$**)]×100% (Formula 8.1)**

Where N_0_ and N represent the average number of colonies in the blank control group and the experimental group, respectively.

**9. Antioxidant Performance Evaluation:**

(1) DPPH Radical Scavenging Assay: Prepare an ethanol solution as the solvent. First, prepare a DPPH solution with a concentration of 0.08 mg/mL using the solvent. Then, place the prepared hydrogel in the DPPH solution and let it stand in the dark at room temperature for 60 minutes. Use an ethanol solution as the blank baseline. Measure the absorbance (Abs) of the treated solution at 517 nm using a UV spectrophotometer. Calculate the DPPH radical scavenging rate using formula 9.1.

$\boldsymbol{DPPH} \boldsymbol{scavenging}\left（ \boldsymbol{\%} \right）\mathbf{=}\frac{\boldsymbol{A}_{\mathbf{0}}\mathbf{-}\boldsymbol{A}_{\mathbf{b}}}{\boldsymbol{A}_{\mathbf{0}}}\boldsymbol{\times100\%}$ **(Formula 9.1)**

Where A_0_ and A_b_ represent the absorbance of the DPPH solution and the DPPH solution after reaction with the sample, respectively.

(2) Hydroxyl Radical (OH·) Scavenging Assay: The hydroxyl radical scavenging assay is based on the Fenton reaction. First, mix 300 μL of the hydrogel with 600 μL of FeSO4 solution (2 mM) and 500 μL of Safranin O solution (360 μg/mL), and incubate the mixture for 10 minutes. Then, add 800 μL of H2O2 solution (6 wt%), and incubate the mixed solution at 55°C for 60 minutes. Use 300 μL of deionized water as the blank control, and use 300 μL and 800 μL of deionized water as the control for the hydrogel and H2O2 solution, respectively. Measure the absorbance (Abs) of the solution at 492 nm using a spectrophotometer. Calculate the hydroxyl radical scavenging rate using formula 9.2.

$\boldsymbol{OH\cdot}\boldsymbol{scavenging}\left（ \boldsymbol{\%} \right）\mathbf{=}\frac{\boldsymbol{A}_{\mathbf{b}}\mathbf{-}\boldsymbol{A}_{\mathbf{0}}}{\boldsymbol{A}_{\mathbf{c}}\mathbf{-}\boldsymbol{A}_{\mathbf{0}}}\boldsymbol{\times100\%}$ **(Formula 9.2)**

Where A_0_, A_b_, and Ac represent the absorbance of the blank control, the experimental group, and the control group solution, respectively.

**10. Evaluation of Conductive Hydrogel in Promoting Chronic Wound Healing in Diabetic Rats:**

Nine healthy male SD rats, weighing 220-250g and 7-8 weeks old, are selected. They are randomly divided into 4 groups, with 3 rats per group and 3 rats per cage. The rats are allowed to freely feed and drink water and are adapted to the environment for one week. After an overnight fast of 12 hours, the male SD rats (250-300g) are intravenously injected with streptozotocin (60 mg/kg body weight) to induce type II diabetes. Blood glucose levels are monitored through tail vein blood. Once the blood glucose level exceeds 16.67 mM, it is considered that the type II diabetes rat model has been successfully established. After successful modeling (i.e., when blood glucose level exceeds 16.67 mM), the rats are anesthetized with 10% (w/v) hydrated chloral (0.3 mL/100 g body weight). The back of each rat is depilated and disinfected, and four square full-thickness wounds (~1*1cm) are formed on the back of each rat. Each wound is inoculated with 50μL of bacterial suspension containing 1×10^8^ cfu/mL of Staphylococcus aureus.

The wounds are divided into four groups as follows:

Blank group: Wounds treated with physiological saline. Control group: Wounds treated with Ag^+^ commercial dressing. Experimental groups: a. GO group: Wounds covered with GO-PAM hydrogel. b. rGO group: Wounds covered with Hep_20_-PDA_0.8_-rGO-PAM hydrogel. c. rGO + ES group: Wounds covered with Hep_20_-PDA_0.8_-rGO-PAM hydrogel, and this group is subjected to direct current stimulation of 100 mv/mm for 1 hour every other day during the healing process. The commercial dressing group changes dressings weekly during the healing process. The wound size is measured at predetermined time intervals (0 days, 1 day, 3 days, 7 days, and 14 days after injury) by tracing the wound border, and the wound healing rate is calculated using formula 10.1.

$Wound healing rate\left（ \% \right）=\frac{S_{0}-S_{n}}{S_{0}}\times100\%$ (Formula 10.1)

Where S_0_ and S_n_ represent the initial wound area and the wound area on the nth day, respectively. On the 14th day, the rats are euthanized, and the wound tissues are collected. The collected tissues are first fixed with 4% paraformaldehyde, embedded in paraffin for H&E staining and Masson staining, and subjected to immunohistochemical analysis.

**11. Results**

**Table S1 Content of various components in conductive hydrogel**

| Hydrogels | PDA/  AM  (wt.%) | Hep/  AM  (wt.%) | GO/  AM  (wt.%) | AM(g) | APS/AM(wt.%) | MBA/AM(wt.%) | TMEDA(μL) | H_2_O  (mL) |
| --- | --- | --- | --- | --- | --- | --- | --- | --- |
| PAM | 0 | 0 | 0 | 2.5 | 5 | 1.2 | 20 | 10 |
| GO-PAM | 0 | 0 | 1 | 2.5 | 5 | 1.2 | 20 | 10 |
| Hep_5_-rGO-PAM | 0 | 5 | 1 | 2.5 | 5 | 1.2 | 20 | 10 |
| Hep_10_-rGO-PAM | 0 | 10 | 1 | 2.5 | 5 | 1.2 | 20 | 10 |
| Hep_20_-rGO-PAM | 0 | 20 | 1 | 2.5 | 5 | 1.2 | 20 | 10 |
| PDA_0.2_-rGO-PAM | 0.2 | 0 | 1 | 2.5 | 5 | 1.2 | 20 | 10 |
| PDA_0.4_-rGO-PAM | 0.4 | 0 | 1 | 2.5 | 5 | 1.2 | 20 | 10 |
| PDA_0.8_-rGO-PAM | 0.8 | 0 | 1 | 2.5 | 5 | 1.2 | 20 | 10 |
| Hep_20_-PDA_0.2_-rGO-PAM | 0.2 | 20 | 1 | 2.5 | 5 | 1.2 | 20 | 10 |
| Hep_20_-PDA_0.4_-rGO-PAM | 0.4 | 20 | 1 | 2.5 | 5 | 1.2 | 20 | 10 |
| Hep_20_-PDA_0.8_-rGO-PAM | 0.8 | 20 | 1 | 2.5 | 5 | 1.2 | 20 | 10 |


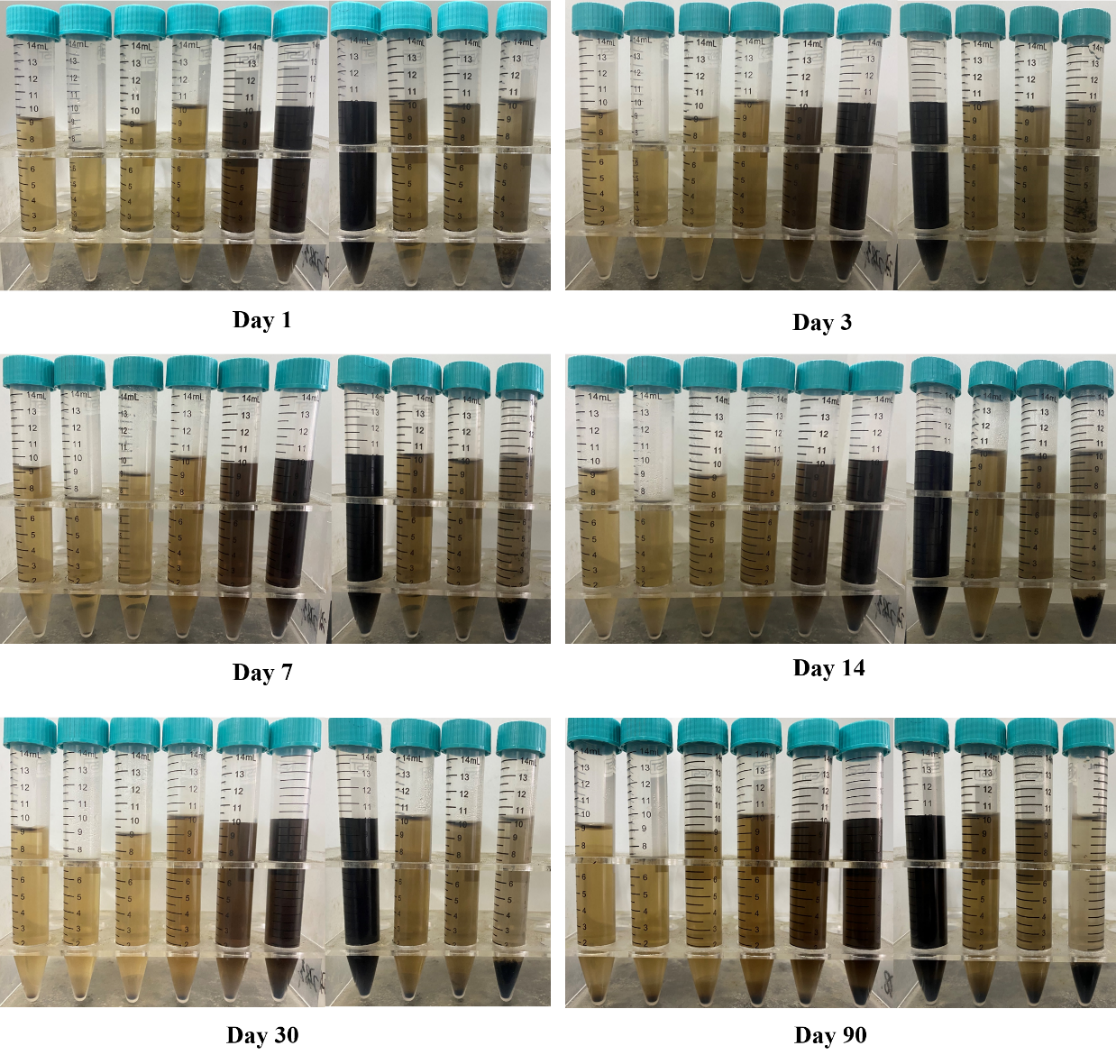


Figure S1. Dispersion stability of different component nanosheets in water (from left to right, GO, Hep_5_-rGO, Hep_10_-rGO, Hep_20_-rGO, Hep_20_-PDA_0.2_-rGO, Hep_20_-PDA_0.4_-rGO, Hep_20_-PDA_0.8_-rGO, PDA_0.2_-rGO, PDA_0.4_-rGO, PDA_0.8_-rGO)


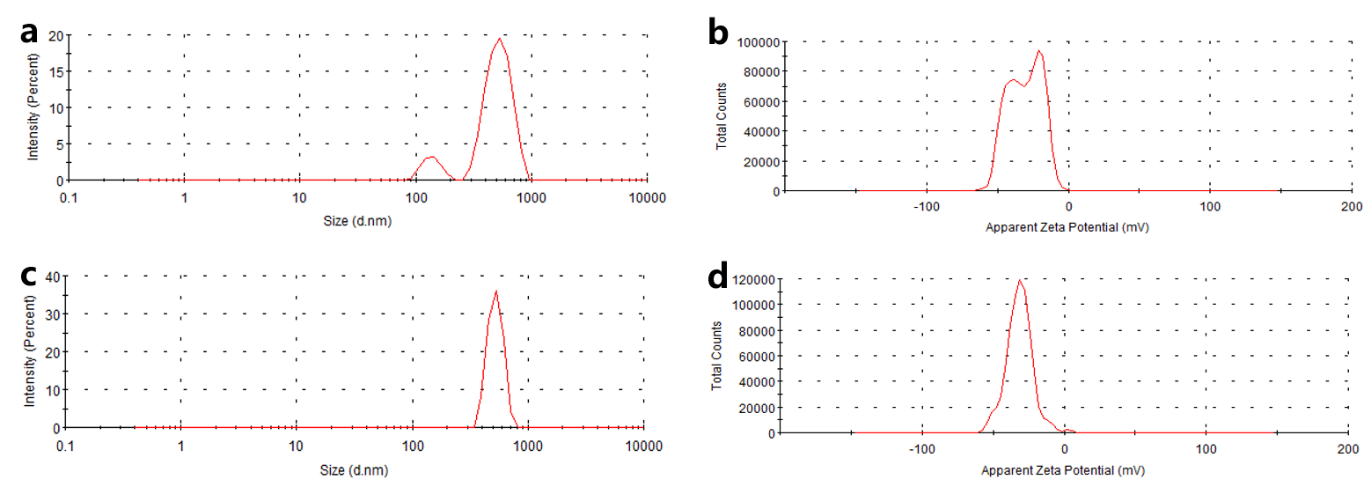


Figure S2 shows the particle size distribution (a) and potential (b) of GO nanosheets in water, as well as the particle size distribution (c) and potential (d) of PDA_0.8_-Hep_20_-rGO nanosheets in water.

**Table S2** Particle size and potential of rGO nanosheets at different ratios.

| **Nano sheets** | **zeta potential in DI water(mV)** | **diameter in DI water(nm)** |
| --- | --- | --- |
| GO | -25.2±0.75 | 595.5±63.8 |
| Hep_5_-rGO | -27.0±1.5 | 687.5±5.4 |
| Hep_10_-rGO | -28.4±0.76 | 706.1±22.6 |
| Hep_20_-rGO | -29.3±1.25 | 763.5±25.8 |
| PDA_0.2_-rGO | -23.3±4.6 | 917.3±57.9 |
| PDA_0.4_-rGO | -28.1±2.5 | 766.1±49.4 |
| PDA_0.8_-rGO | -30.9±0.6 | 706.1±13.1 |
| Hep_20_-PDA_0.2_-rGO | -31.7±0.4 | 879.7±32.2 |
| Hep_20_-PDA_0.4_-rGO | -32.1±2.5 | 788.9±59.7 |
| Hep_20_-PDA_0.8_-rGO | -33±0.60 | 781.1±26.4 |
